# The wake-promoting drug modafinil stimulates specific hypothalamic circuits to promote adaptive stress responses in an animal model of PTSD

**DOI:** 10.1038/tp.2016.172

**Published:** 2016-10-11

**Authors:** S Cohen, G Ifergane, E Vainer, M A Matar, Z Kaplan, J Zohar, A A Mathé, H Cohen

**Affiliations:** 1 Anxiety and Stress Research Unit, Beer-Sheva Mental Health Center, Ministry of Health, Faculty of Health Sciences, Ben-Gurion University of the Negev, Beer-Sheva, Israel; 2 Department of Psychology, Ben-Gurion University of the Negev, Beer-Sheva, Israel; 3 Headache Clinic, Department of Neurology, Soroka Medical Centre, Ben-Gurion University of the Negev, Beer- Sheva, Israel; 4 Division of Psychiatry, The Chaim Sheba Medical Center, Ramat-Gan, Israel; 5 Sackler Medical School, Tel-Aviv University, Tel-Aviv, Israel; 6 Karolinska Institutet - Clinical Neuroscience, Karolinska University Hospital Huddinge, Stockholm, Sweden

## Abstract

Pharmacotherapeutic intervention during traumatic memory consolidation has been suggested to alleviate or even prevent the development of posttraumatic stress disorder (PTSD). We recently reported that, in a controlled, prospective animal model, depriving rats of sleep following stress exposure prevents the development of a PTSD-like phenotype. Here, we report that administering the wake-promoting drug modafinil to rats in the aftermath of a stressogenic experience has a similar prophylactic effect, as it significantly reduces the prevalence of PTSD-like phenotype. Moreover, we show that the therapeutic value of modafinil appears to stem from its ability to stimulate a specific circuit within the hypothalamus, which ties together the neuropeptide Y, the orexin system and the HPA axis, to promote adaptive stress responses. The study not only confirms the value of sleep prevention and identifies the mechanism of action of a potential prophylactic treatment after traumatic exposure, but also contributes to understanding mechanisms underlying the shift towards adaptive behavioral response.

## Introduction

Recurring memories of a traumatic event are a fundamental feature of posttraumatic stress disorder (PTSD) and underlie many aspects of its clinical manifestation, including intrusive thoughts, physiological hyperarousal, avoidance behavior and emotional numbing.^1^ It is well established that, following initial encoding, memories remain temporarily vulnerable to disruption^2^ until they are consolidated. Emerging evidence suggests that a pharmacological secondary prevention can alter the brain mechanisms that regulate the formation, storage and retrieval of traumatic memories.

Convergent evidence indicates that sleep serves as an off-line period in which newly encoded hippocampus-dependent memories are gradually adapted to pre-existing knowledge networks.^3, 4^ Sleep that follows learning, independent of the time of the day, enhances the consolidation of newly acquired memory traces^3, 5^ through an active reorganization of representations, whereas acute sleep deprivation may disrupt this process and impair retrieval functions.^6^ We recently reported that depriving rats of sleep for 6 h following exposure to a stressful, traumatic-like event significantly reduces the prevalence of their PTSD-like behaviors and impairs the formation and consolidation of hippocampus-dependent traumatic memories—a mechanism possibly relevant to the development of PTSD.^7^ The next logical step is to examine, in an established animal model of PTSD, whether pharmacologically disrupting sleep in the aftermath of a stressogenic experience can alleviate or altogether prevent PTSD-related symptoms. To this end, in the current study, we tested whether early treatment with a sleep-depriving pharmacological agent can mimic the anxiolytic effects of a 6 h sleep deprivation following exposure to predator-scent stress (PSS)—an established rat model of PTSD^8, 9, 10, 11^—and began exploring the underlying mechanisms. We chose to use modafinil [(RS)-2-(diphenylmethylsulfinyl)acetamide] although the mechanism of action of this drug is yet unknown,^12^ it has been approved by the FDA for treating excessive daytime sleepiness associated with narcolepsy, shift work sleep disorder and obstructive sleep apnea/hypopnea syndrome.

## Materials and methods

All the procedures were carried out under strict compliance with ethical principles and guidelines of the NIH Guide for the Care and Use of Laboratory Animals. All the treatment and testing procedures were approved by the Animal Care Committee of Ben-Gurion University of the Negev, Israel [IL-29-04-2015].

A sample of 406 adult male Sprague Dawley rats, weighing 180–220 g, was used. The rats were housed, three per cage, in a vivarium with a stable temperature, a 12:12 light–dark cycle (lights off at 1900 h; luminous emittance during the light phase: 200G50 lux), with unlimited access to food and water. The animals were assigned and randomized as described in the Supplementary Materials and Methods. All the rats were maintained under this regime for a 1-week habituation period before the experiment began. All the procedures were performed during the resting phase of the rats, between 0830 and 1200 h. During this period, namely, several hours before the onset of the diurnal plasma corticosterone peak, corticosterone levels remained low and stable.^13^ Indeed, the peak of plasma corticosterone levels occurred 2 h before the beginning of the activity phase, that is, at about 1700 h. Thus, we assume that the circadian influence on corticosterone level and behavior did not interfere with the interpretation of our results.

### Predator-scent stress

The rats were individually placed on well-soiled cat litter, which was used by a cat for 2 days and sifted for stools. The rats were exposed to the litter for 10 min in a plastic cage (inescapable exposure) placed on a yard paving stone in a closed environment. Sham-PSS was administered under similar conditions, but the rats were exposed to a fresh, unused cat litter.^8, 9, 10^

### Drugs

Modafinil (Cephalon, Frazer, PA, USA) was dissolved in distilled water and administered intraperitoneally (150 or 350 mg kg^−1^, as indicated). Almorexant—(2R)-2-[(1S)-6,7-Dimethoxy-1-[2-(4-trifluoromethyl-phenyl)-ethyl]-3,4-dihydro-1H-isoquinolin-2-yl]-*N*-methyl-2-phenyl-acetamide (150 mg kg^−1^)—was synthesized at ChemBo Pharma (Kowloon, Hong Kong) according to the literature.^14^ Almorexant was dissolved in saline and was injected intraperitoneally. The Y1R-selective argininamide derivative BIBO3304—(R)-N-[[4-(aminocarbonylaminomethyl)-phenyl]methyl]-N2-(diphenylacetyl)-argininamide trifluoroacetate^15^—was purchased from Tocris Bioscience (Kfar-Saba, Israel). A 10 μl volume of BIBO3304 or distilled water was infused into each nare with pipetteman and disposable plastic tip under light isoflurane anesthesia (Intranasal administration). Mifepristone was purchased from Sigma-Aldrich (Rehovot, Israel), and was dissolved in a dose of 7.5 mg (approximately 30 mg kg^−1^) in 0.5 ml propylene glycol as a vehicle. All the drugs were prepared fresh before use. The drug doses were chosen on the basis of previous studies.^16, 17, 18^

### Behavioral measurements

#### Elevated plus-maze

The maze was a plus-shaped platform with two opposing open arms and two opposing closed arms (closed arms surrounded by 14-cm-high opaque walls on three sides).^12^ Rats were placed on the central platform, facing an open arm, and were allowed to freely explore the maze for 5 min. Each test was videotaped and the behavior of the rat was subsequently scored by an independent observer. Arm entry was defined as entering an arm with all four paws. At the end of the 5 min test period, the rat was removed from the maze, the floor was wiped with a damp cloth, and any faecal boluses were removed. Behaviors assessed were time spent (duration) in open and closed arms and on the central platform; number of entries to the open and closed arms; and total exploration, which was calculated as the number of entries into any of the arms and was used to distinguish between impaired exploratory behavior, exploration limited to closed arms (avoidance), and free exploration. An ‘Anxiety Index', which integrates the elevated plus-maze (EPM) behavioral measures, was calculated as follows:


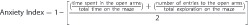


#### Acoustic startle response

Startle responses were measured by using two ventilated startle chambers (SR-LAB system, San Diego Instruments, San Diego, CA, USA). The SR-LAB calibration unit was used routinely to ensure consistent stabilimeter sensitivity between the test chambers and over time. Each Plexiglas cylinder rested on a platform inside a soundproof, ventilated chamber. Movement inside the cylinder was detected by a piezoelectric accelerometer below the frame. Sound levels within each test chamber were measured routinely with a sound level meter (Radio Shack) to ensure consistent presentation. Each test session began with a 5-min acclimatization period to background white noise of 68 dB, followed by 30 acoustic startle trial stimuli presented in 6 blocks (110 dB white noise of 40 ms duration and 30 or 45 s inter-trial interval). Two behavioral parameters were assessed: (a) the mean startle amplitude (averaged over all 30 trials); and (b) the percent of startle habituation to repeated presentation of the acoustic pulse. For the latter, the percent change was calculated between the response to the first and last (sixth) blocks of sound stimuli, as follows:


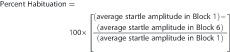


According to the EPM and acoustic startle response (ASR) measurements, the response of each rat to the PSS or Sham-PSS exposure was classified as an extreme, minimal, or partial behavioral response (EBR, MBR, and PBR, respectively; see below).^9^

### The cut-off behavioral criteria model

The classification of individuals according to the degree to which their individual behavior was affected by a stressor is based on the premise that extremely compromised behavior in response to the priming trigger is not conducive to survival and is thus inadequate and maladaptive, representing a pathological degree of response.^9, 10, 11, 19^

### Immunohistochemistry

Twenty-four hours after the behavioral tests (that is, on Day 8), animals were deeply anesthetized and perfused transcardially with cold 0.9% physiological saline followed by 4% paraformaldehyde (Sigma-Aldrich, Rehovot, Israel) in 0.1 M phosphate buffer. Brains were quickly removed, postfixed in the same fixative for 12 h at 4 °C, cryoprotected overnight (30% sucrose in 0.1 M phosphate buffer at 4 °C), and then frozen and stored at −80 °C. Serial coronal sections (10 μm) were performed with a cryostat (Leica CM 1850; Leica Microsystems, Wetzlar, Germany) and mounted on coated slides.

### Staining

Sections were air-dried and washed three times in phosphate-buffered saline (PBS) containing Tween 20 (PBS/T) (Sigma-Aldrich). They were then incubated for 60 min in a blocking solution (normal goat serum in PBS) and then overnight at 4 °C with the primary antibodies against NPY (mouse monoclonal anti-NPY antiserum (1:500), product code: sc-133080, Santa Cruz Biotechnology, Heidelberg, Germany), ORX-A and ORX-B (rabbit polyclonal anti-ORX-A antiserum (1:500), product code: ab-6214, and rabbit polyclonal anti-ORX-B antiserum (1:500), product code: ab-170999, Abcam, Cambridge, UK). After three washes in PBS/T, the sections were incubated for 2 h in DyLight-488-labeled goat-anti-rabbit IgG or in Dylight-594 goat anti-mouse IgG (1:250; KPL, Gaithersburg, MD, USA) in PBS containing 2% normal goat or horse serum. The sections were then washed and mounted with a mounting medium (Vectastain; Vector Laboratories, Burlingame, CA, USA). Sections from the brains of different groups of rats were processed at the same time and under identical conditions to ensure reliable comparisons and to maintain stringency in tissue preparation and staining conditions. Control staining was performed in the absence of the primary antibodies. Additionally, secondary fluorescent labels were swapped to test for cross-reactivity, and sections were incubated without primary antibodies to test for non-specific binding of the secondary antibodies.

#### Relative quantitative analysis of NPY-, ORX-A- and ORX-B-immunoreactivity

Brain sections showing NPY-, ORX-A and ORX-B-ir in the PVN (–1.4 to –2.4 mm), arcuate hypothalamic nucleus (ARC) (–2.4 to –3.4 mm), PVTN (–1.8 to –2.4 mm) or Pef (–2.4 to –3.0 mm) (all coordinates relative to bregma) were subjected to image analysis. Each brain region was defined under the microscope according to cytoarchitectural landmarks.^14^ NPY- and ORX-ir were measured in a 50 000 μm^2^ area in each area of interest (AOI) and were digitized by using microscopic images (Leica microscope DM4500B) and a DFC340FX digital imaging camera (Leica). Measurements were taken from predetermined fields in each subregion from both brain hemispheres. The density of fibers and cells expressing NPY- and ORXs-ir in each area was determined with the Leica LAS software (version 3.8). To compensate for background staining levels and control for variations in the overall illumination levels between images, the average pixel density of two regions that, presumably, contained only nonspecific staining (that is, in areas that are near each AOI that is not thought to contain NPY/ORXs) was determined within each captured image, and this value was subtracted from all density measurements performed on that image.^15, 16^ Results are expressed as the average staining intensity and are presented as the mean ± standard error of the mean (s.e.m.). The data from all animals in each group were pooled separately for each brain region, and the mean±s.e.m. was calculated.

### Animal sacrifice and blood sampling

Rats were decapitated with a guillotine. Situational stress was minimized by thoroughly cleaning the area between sacrifices and removing the bodies. Trunk blood was collected, left at room temperature for 1 h and then centrifuged at 1000 *g* for 10min at 4 °C. Serum was collected and stored at –80 °C.

### Measurement of serum corticosterone

Corticosterone was measured with a DSL-10-81000 ELISA kit (Diagnostic Systems Laboratories, Webster, TX, USA) according to the instructions of the manufacturer, by a person blind to the experimental procedures. All the samples were measured in duplicates.

### Statistical analyses

For the behavioral and molecular results, statistical analyses were performed with a two-way analysis of variance with PSS exposure (PSS vs Sham-PSS) and treatment (modafinil vs vehicle) as the independent factors. For the molecular effects of pharmacologically manipulating NPY, ORXs or corticosterone levels before the modafinil/vehicle treatment, statistical analyses were performed with a one-way analysis of variance. Bonferroni tests were used to examine the differences between individual groups. In addition, the behavioral data were transformed to percentage by using the cut-off behavioral criteria model: the prevalence of affected rats as a function of rat group was tested by using cross-tabulation and nonparametric chi-squared tests. All nonparametric analyses were performed on raw data (and not on percentage).

## Results

### Modafinil reversibly promotes wakefulness in rats

We first aimed at determining the optimal dose of modafinil required to increase wakefulness in Sprague Dawley rats. On the basis of the literature^20^ and on the pharmacodynamics of modafinil (100 mg kg^−1^: peak concentration=10.7 μg ml^−1^ at 279 min; half-life=277 min), we administered either 0 (vehicle), 150 or 350 mg kg^−1^ modafinil intraperitoneally to rats during their resting (light) phase. As an indicator of wakefulness, we used both an automated system to continuously record locomotor activity throughout the 24 h period immediately following the administration and visual observations (Supplementary Figure S1a). We found a significant main effect for treatment (F(2,9)=8.98, *P*<0.008, repeated-measures analysis of variance), such that, when administered at 350 mg kg^−1^—but not at 150 or 0 mg kg^−1^—modafinil significantly increased locomotor activity immediately following administration (Supplementary Figure S1b). The cumulative locomotor activity duration of rats treated with 350 mg kg^−1^ modafinil appeared to subside completely within 6 h (Supplementary Figure S1c). No significant differences in locomotor activity were found between the groups during the active phase, indicating that the acute effect of modafinil was reversible. As stereotyped behavior was not observed, modafinil was administered at 350 mg kg^−1^ in all the subsequent experiments.

### Modafinil administration following a stressogenic event attenuates subsequent behavioral stress responses

Using our validated animal model of PTSD, we next assessed the long-term behavioral effects of a single injection of either modafinil (350 mg kg^−1^) or vehicle to rats 30 min after exposing them for 15 min to PSS or to Sham-PSS (that is, to used or unused cat litter, respectively). Seven days later, we quantified the behavior of these rats in two well-established, stress-related paradigms—the EPM and the ASR. These paradigms measure anxiety-like, fearful, avoidant and hyper-vigilant/hyper-alert behaviors, which parallel the different aspects of traumatic stress-induced behaviors in humans.^21^ Exploratory behavior in the EPM served as our main framework for assessing the overall stress-related behavior of the rats, whereas the magnitude of response and the habituation to the acoustic stimulus in the ASR paradigm were used to quantify hyper-alertness,^10^ specifically. Finally, we used the results of these paradigms in a statistically validated cut-off behavioral criteria model^8, 9, 10, 11^ (Supplementary Figure S2) to functionally classify rats according to their overall stress-related behavior.

#### Elevated plus maze

**Figure 1:**
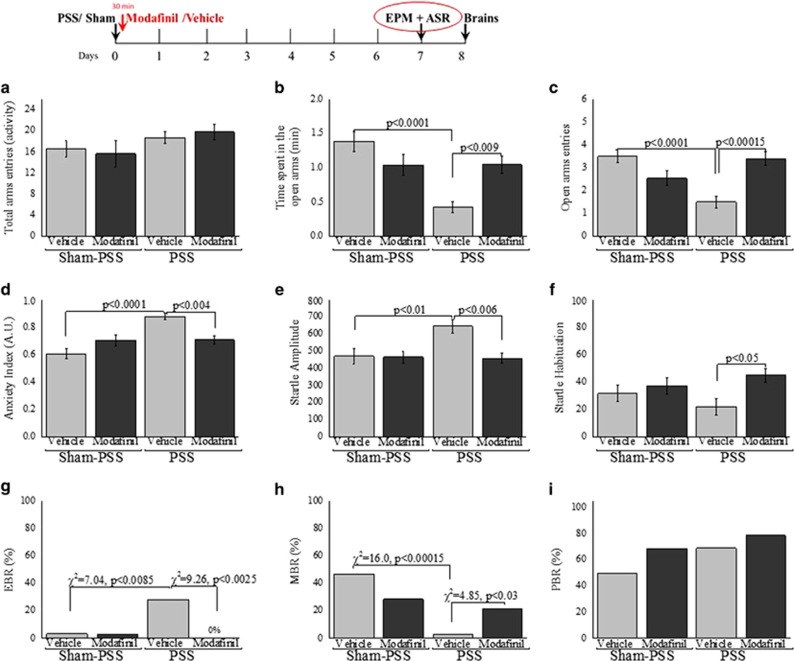
Modafinil administration following a traumatic event attenuates subsequent behavioral stress responses. The experimental protocol is depicted on the top panel. The rats were exposed for 10 min to predator-scent stress (PSS) or to Sham-PSS on day 0, injected 30 min later with modafinil (350 mg kg^−1^; PSS-exposed: *n*=28; Sham-PSS-exposed: *n*=35) or vehicle (PSS-exposed: *n*=32; Sham-PSS-exposed: *n*=30) intraperitoneally, tested in the elevated plus maze (EPM) and acoustic startle response (ASR) on day 7, and killed on day 8 for brain removal and analysis. (**a**) Overall activity in the EPM, as reflected in the total number of entries to the open and closed arms. (**b**) Time spent in the open arms of the EPM (two-way analysis of variance (ANOVA): exposure (main effect): F(1,121)=13.3, *P*<0.0004; exposure × treatment interaction: F(3,121)=13.54, *P*<0.0004). (**c**) Number of entries to the open arms of the EPM (two-way ANOVA: exposure × treatment interaction: F(1,121)=23.6, *P*=0.0001). (**d**) Overall Anxiety Index, which integrates the measured behavioral measures on the EPM (two-way ANOVA: exposure (main effect): F(1,121)=42.7, *P*<0.0009; exposure × treatment interaction (F(1,121)=16.4, *P*<0.001)). (**e**) Startle amplitude in the ASR paradigm (two-way ANOVA: exposure: F(1,121)=4.86, *P*<0.03; treatment (main effect): F(1,121)=6.2, *P*<0.015; exposure × treatment interaction: F(1,121)=5.6, *P*<0.02). (**f**) Percentage of startle habituation in the ASR paradigm (two-way ANOVA: treatment (main effect): F(1,121)=5.8, *P*<0.02); (**g**) Prevalence of extreme post-stress behavioral responses (EBRs; in percentages; Pearson *χ*^2^=20.2, df=3, *P*<0.0002). (**h**) Prevalence of minimal post-stress behavioral responses (MBRs; in percentages; Pearson *χ*^2^=16.2, df=3, *P*<0.0015). (**i**) Prevalence of partial post-stress behavioral responses (PBRs; in percentages). Following a single 10-min exposure to PSS, modafinil administration significantly decreases anxiety-like behaviors, as compared with vehicle administration. Concomitantly, the prevalence of EBRs following PSS exposure is markedly lower in rats treated with modafinil than in rats treated with vehicle. Bars represent group mean±s.e.m. (**a–f**) or percentages (**g–i**).

All the rats showed similar overall activity in the EPM (Figure 1a). However, in the vehicle-treated groups, rats exposed to PSS spent significantly less time in the open arms of the EPM (Figure 1b), entered the open arms significantly less frequently (Figure 1c), and exhibited a significantly higher overall anxiety index (Figure 1d), as compared with rats exposed to Sham-PSS (*P*<0.001 for each parameter). Modafinil ameliorated the behavioral effects of PSS exposure; rats treated with modafinil following PSS exposure spent significantly more time in the open arms of the maze (*P*<0.009), entered the open arms more often (*P*<0.00015), and exhibited a significantly lower anxiety index (*P*<0.004) than rats treated with vehicle following the PSS exposure (Figure 1b). Importantly, in rats treated with modafinil following PSS exposure, the overall time spent in the open arms and the number of entries into the open arms were similar to those of (modafinil- or vehicle-treated) rats that were exposed to Sham-PSS. In addition, in the Sham-PSS groups, no differences were observed between the modafinil-treated and the vehicle-treated rats, indicating that modafinil had no intrinsic effects on behavior. Taken together, these findings indicate that a single injection of modafinil following exposure to a stressogenic event significantly attenuates anxiety-like behaviors.

#### Acoustic startle response

In the vehicle-treated groups, the rats exposed to PSS showed hyperarousal (namely, a significantly enhanced ASR; *P*<0.006), as compared with the rats exposed to Sham-PSS (Figure 1e). Similar to the results of the EPM paradigm, modafinil ameliorated the behavioral effects of PSS exposure in the ASR paradigm, as rats treated with modafinil following the exposure showed a significantly lower mean startle amplitude (*P*<0.01; Figure 1e) and a significantly improved startle habituation (*P*<0.05; Figure 1f), as compared with rats treated with vehicle following PSS exposure. Also similar to the results of the EPM, no differences were observed between the modafinil-treated rats and the vehicle-treated rats in the Sham-PSS groups, indicating that modafinil had no intrinsic effects on behavior.

#### Classification according to the cut-off behavioral criteria model

PTSD-like extreme behavioral responders (EBRs; Figure 1g) were significantly more prevalent in rats exposed to PSS and treated with vehicle (namely, 9/32 rats) than in rats exposed to Sham-PSS and treated with vehicle (1/30 rats; *χ*^2^=7.04, *P*<0.0085) and then rats exposed to PSS and treated with modafinil (0/30 rats; *χ*^2^=9.26, *P*<0.0025). Concomitantly, in the PSS-exposed groups, the prevalence of ‘stress-free' minimal behavioral responders (Figure 1h) was markedly higher in rats treated with modafinil than in the rats treated with vehicle (6/28 and 1/32 rats, respectively; *χ*^2^=4.85, *P*<0.03). Taken together, these analyses indicate that treating rats with modafinil following exposure to a stressogenic event induces a significant shift toward a less-extreme behavioral disruption, suggesting that it confers some degree of resistance to the trauma-related sequelae.

### Modafinil administration following a stressogenic event increases circulating corticosterone levels

**Figure 2:**
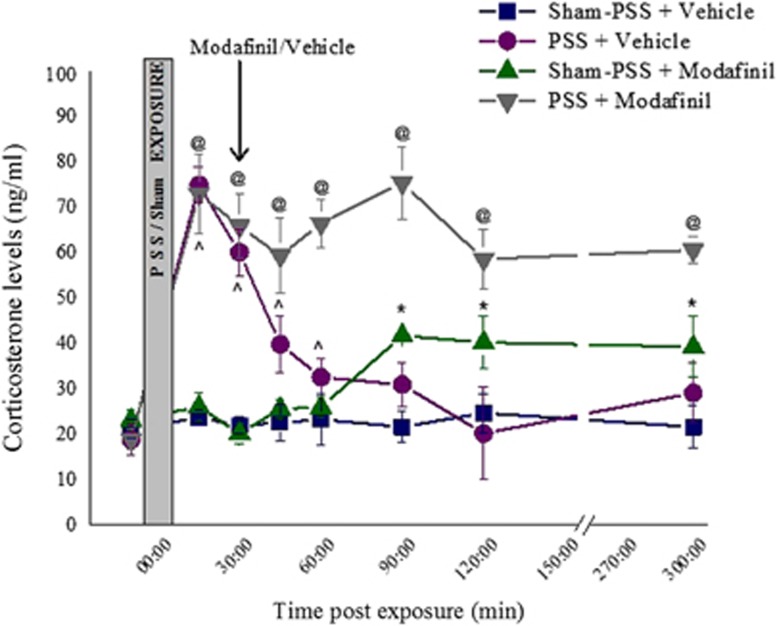
Modafinil administration following a traumatic event increases circulating corticosterone levels. The rats were exposed for 10 min to predator-scent stress (PSS) or to Sham-PSS and were intraperitoneally injected, 30 min later, with modafinil (350 mg kg^−1^) or with vehicle. Circulating corticosterone levels were measured before or 15, 30, 45, 60, 90, 120 or 300 min after the exposure to PSS or Sham-PSS (*n*=96 overall, three rats from each group in each time point). The modafinil treatment modulated circulating corticosterone levels in rats exposed to PSS and in rats exposed to Sham-PSS (two-way analysis of variance: group (main effect): F(3,64)=110.3, *P*<0.0001; time (main effect): F(7,64)=14.7, *P*<0.0001; group × time interaction: F(21,64)=8.93, *P*<0.001). ^@^*P*<0.0001 vs PSS–modafinil baseline level. ^^^*P*<0.01 vs PSS–vehicle baseline level. *P<0.001 vs Sham-PSS+modafinil baseline level. Data points represent group means±s.e.m.

We hypothesized that the mechanism by which modafinil attenuates stress-related behavior after exposure to PSS involves glucocorticoids. To test this hypothesis, we examined whether administering modafinil soon after exposure to PSS modifies the activation of the hypothalamic-pituitary-adrenal (HPA) axis, as reflected in circulating corticosterone levels. In these experiments, we exposed rats to PSS or to Sham-PSS and, 30 min after the exposure, we injected either modafinil (350 mg kg^−1^) or vehicle intraperitoneally. We monitored circulating corticosterone levels before exposure (baseline) and 15, 30, 45, 60, 90, 120 and 300 min following exposure. Bonferroni *post hoc* comparisons indicated that rats who were exposed to PSS and then treated with vehicle showed an immediate increase in corticosterone levels—as expected from such a stressful exposure—which peaked 15 min following exposure and then declined steeply and reached basal levels at 60 min following exposure. In marked contrast, the rats who were exposed to PSS and then treated with modafinil showed a similar immediate increase in corticosterone levels, but these levels remained significantly elevated and did not reach baseline levels throughout the 5 h of the experiment. The rats who were exposed to Sham-PSS and then treated with modafinil displayed significantly higher circulating corticosterone levels 60 min following the injection (90 min after PSS exposure), as compared with the corticosterone levels of rats before and 15, 30, 45 and 60 min following the Sham-PSS exposure (*P*<0.025 for each time point; Figure 2). These findings imply that the manner by which modafinil modulates circulating corticosterone levels is different in rats exposed to PSS and in rats exposed to Sham-PSS, although modafinil had an intrinsic effect on both the groups.

### Modafinil increased immunoreactivity of orexin-A, orexin-B and NPY in the hypothalamus

To gain insights into the molecular mechanism by which modafinil attenuated the posttraumatic stress responses, we killed rats at the beginning of their inactive phase, 24 h following the behavioral tests (that is, 8 days after they were exposed to PSS or to Sham-PSS) and collected their brains for immunohistochemical staining. We used a computer-assisted imaging system to quantitatively analyze the density of immunoreactive orexin-A, orexin-B and NPY fibers and cells in different hypothalamic areas.

#### Anatomical interactions between orexins and NPY

In all the treatment groups, the immunolabeling patterns of orexins and NPY in the hypothalamus were generally consistent with those described previously,^22, 23^ with a dense network of NPY-immunoreactive fibers and cells observed throughout the areas of the hypothalamus in which orexin cells were located. A double-immunofluorescence protocol revealed that the majority of ORX-A-immunoreactive and ORX-B-immunoreactive cells in the hypothalamus are co-expressed with NPY-immunoreactive cells, whereas some NPY-immunoreactive cells are not co-expressed with ORX-A-immunoreactive or with ORX-B-immunoreactive.

In the vehicle-treated groups, rats who were exposed to PSS exhibited a significantly lower density of ORX-A-immunoreactive, ORX-B-immunoreactive and NPY-immunoreactive cells and fibers, as compared with rats exposed to Sham-PSS, in the PVN (*P*<0.025, *P*<00002 and *P*<0.0001, respectively; Supplementary Figures S2a, e and i), ARC (*P*<0.0015, *P*<0.0001 and *P*<0.0001, respectively; Supplementary Figures S2b, f and j), PVTN (*P*<0.035, *P*<0.01 and *P*<0.002, respectively; Supplementary Figures S2c, g and k), and PeF (*P*<0.0055, *P*<0.05 and *P*<0.0001, respectively; Supplementary Figures S2d, h and l). The PSS-induced decrease in immunoreactivity was sensitive to treatment with modafinil following the PSS exposure, as the density of ORX-A-immunoreactive and NPY-immunoreactive cells and fibers was significantly higher in rats treated with modafinil than in rats treated with vehicle in the PVN (*P*<0.04 and *P*<0.0001, respectively), ARC (*P*<0.0015 and *P*<0.0001, respectively), PVTN (*P*<0.02 and *P*<0.0001, respectively) and PeF (*P*<0.002 and *P*<0.0001, respectively). In addition, in the modafinil-treated groups, the rats who were exposed to PSS, as compared with those who were exposed to Sham-PSS, demonstrated a significantly higher density of ORX-B-immunoreactive cells and fibers in the PVN, ARC and PVTN (*P*<0.0001, *P*<0.04 and *P*<0.015, respectively) and a significantly higher density of NPY-immunoreactive cells and fibers in the PVTN and PeF (*P*<0.05 and *P*<0.003, respectively). Taken together, these findings suggest that modafinil acts, at least in part, by activating the NPYergic and the ORXergic systems, as can be observed at least 8 days following the PSS exposure.

### The NPY-Y1 receptor and the ORX receptors are required for the anxiolytic effects of modafinil

To test the extent to which the observed anxiolytic effects of modafinil are directly associated with the NPY-Y1 receptor (NPY-Y1R), the ORX receptors (ORXRs) or the glucocorticoid receptors (GRs), we injected rats with either (a) the NPY-Y1R antagonist BIBO3304; (b) the dual orexin receptor antagonist almorexant; (c) the GR antagonist mifepristone or (d) vehicle, in each case with or without modafinil treatment. Four different control groups were used, grouped by path of administration and type of solvent used. Because no statistical differences were observed between all control groups, we combined the control groups together. We performed the antagonist injections 10 min before, or, for mifepristone, 5 min following exposure to PSS and injected modafinil intraperitoneally 30 min following exposure to PSS. Behavior was evaluated in the EPM and ASR paradigms 7 days following the exposure to PSS. Then, 1 day later, the rats were killed, their brains collected, and the hypothalamic NPY-immunoreactive, ORX-A-immunoreactive and ORX-B-immunoreactive were quantified.

**Figure 3:**
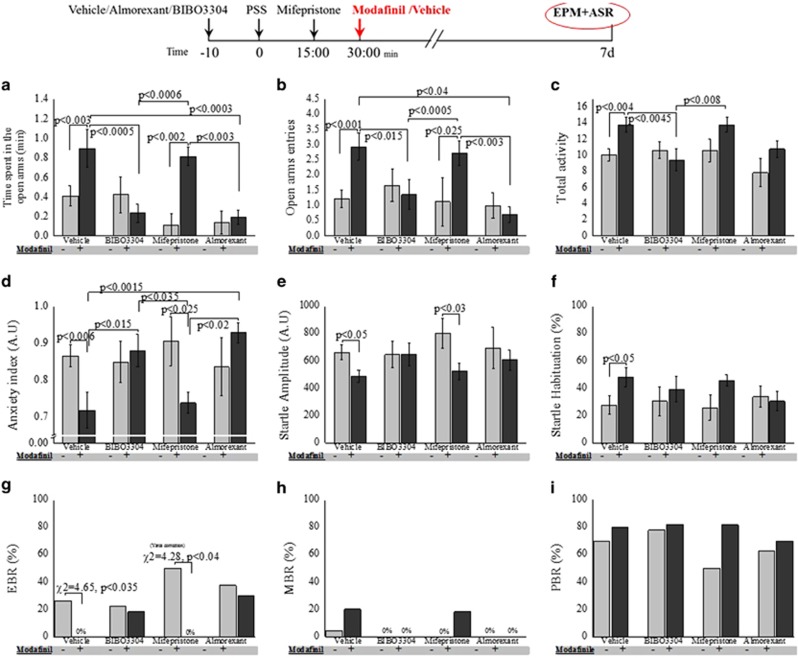
The NPY-Y1 receptor and the ORX receptors are necessary for the anxiolytic effects of modafinil. The experimental protocol is depicted on the top panel. The rats were intraperitoneally injected with either vehicle, BIBO3304 (an NPY-Y1 receptor antagonist), or almorexant (a dual orexin receptor antagonist), and then, 30 min later, they were exposed to predator-scent stress (PSS) for 10 min. Alternatively, the rats were injected with mifepristone (a glucocorticoid receptor antagonist) 5 min after the PSS exposure. Next, 30 min after the PSS exposure, all the rats were intraperitoneally injected either with modafinil (350 mg kg^−1^; vehicle–modafinil: *n*=15; BIBO3304–modafinil: *n*=11; mifepristone–modafinil: *n*=11; almorexant–modafinil: *n*=8) or with vehicle (vehicle–vehicle: *n*=23; BIBO3304–vehicle: *n*=9; mifepristone–vehicle: *n*=8; almorexant–vehicle: *n*=10). Finally, all the rats were tested in the elevated plus maze (EPM) and acoustic startle response (ASR) paradigm on day 7. (**a**) Overall activity in the EPM, as reflected in the total number of entries to the open and closed arms (two-way analysis of variance (ANOVA): blockage: F(3,87)=3.0, *P*<0.04; treatment: F(3,87)=7.0, *P*<0.01). (**b**) Time spent in the open arms of the EPM (two-way ANOVA: blockage: F(3,87)=5.1, *P*<0.003; treatment: F(1,87)=6.9, *P*<0.015; blockage × treatment interaction: F(3,87)=3.9, *P*<0.015). (**c**) Number of entries to the open arms of the EPM (two-way ANOVA: blockage: F(3,87)=2.9, *P*<0.04; treatment: F(1,87)=4.3, *P*<0.045; blockage × treatment interaction: F(3,87)=3.2, *P*<0.03). (**d**) Anxiety index, which integrates the measured EPM behavioral measures (two-way ANOVA: blockage × treatment interaction: F(3,87)=3.7, *P*<0.015). (**e**) Startle amplitude in the ASR paradigm (two-way ANOVA: treatment: F(1,87)=5.4, *P*<0.025). (**f**) Percentage of startle habituation in the ASR paradigm (NS). (**g**) Prevalence of extreme post-stress behavioral responses (EBRs; in percentages; Pearson *χ*2: vehicle–vehicle vs vehicle–modafinil: *χ*2=4.65, *P*<0.035; mifepristone–vehicle vs mifepristone–modafinil: *χ*2=4.28, *P*<0.04). (**h**) Prevalence of minimal post-stress behavioral responses (MBRs; in percentages; NS). (**i**) Prevalence of partial post-stress behavioral responses (PBRs; in percentages; NS). Administering either BIBO3304 or almorexant before PSS exposure completely prevented the ability of modafinil to decrease the prevalence of the PSS-induced PTSD-like phenotype, whereas administering mifepristone had no significant influence on the behavioral response of modafinil. Bars represent group means±s.e.m. (**a–f**) and percentages (**g–i**).

Administrating either the NPY-Y1R antagonist BIBO3304 or the ORXRs antagonist almorexant prevented the anxiolytic effects of modafinil in rats exposed to PSS. In the EPM, rats who were exposed to PSS and treated with BIBO3304 or almorexant (in addition to modafinil) spent significantly less time in the open arms (*P*<0.0005 and *P*<0.0003, respectively; Figure 3a), entered the open arms significantly less (*P*<0.015 and *P*<0.0005, respectively; Figure 3b) and exhibited a significantly higher anxiety index (*P*<0.015 and *P*<0.0015, respectively, Figure 3d) than their vehicle-treated counterparts. The total activity in the maze was significantly lower in rats treated with vehicle than in rats treated with modafinil (*P*<0.004, Figure 3c). The GR antagonist mifepristone, injected at pharmacologically active doses before modafinil treatment, had no significant influence on the anxiolytic effects of modafinil in the EPM. In the ASR paradigm, administering either BIBO3304 or almorexant completely prevented the ability of modafinil to decrease the PSS-induced hyperarousal, whereas administering mifepristone had no significant influence on the hyperarousal response of modafinil (Figures 3e and f). The cut-off behavioral criteria classification (Figures 3g–i) revealed that a pre-injection of either vehicle or mifepristone did not affect the protective effects of modafinil, as none of these rats displayed a PTSD-like EBR (Figure 3g). In marked contrast, a pre-injection of either BIBO3304 or almorexant completely prevented the protective effects of modafinil, as 18.2% of the rats pre-injected with BIBO3304 and 30.0% of the rats pre-injected with almorexant displayed an EBR. In fact, the prevalence of EBRs in these two groups did not differ from the prevalence of EBRs in control rats, who were injected with vehicle instead of modafinil. These findings suggest that the activation of both ORXRs and the NPY-Y1R is required for the anxiolytic effects of modafinil given shortly after a stressogenic exposure to take place, whereas endogenous GC signaling does not appear to be functionally or directly involved.

**Figure 4:**
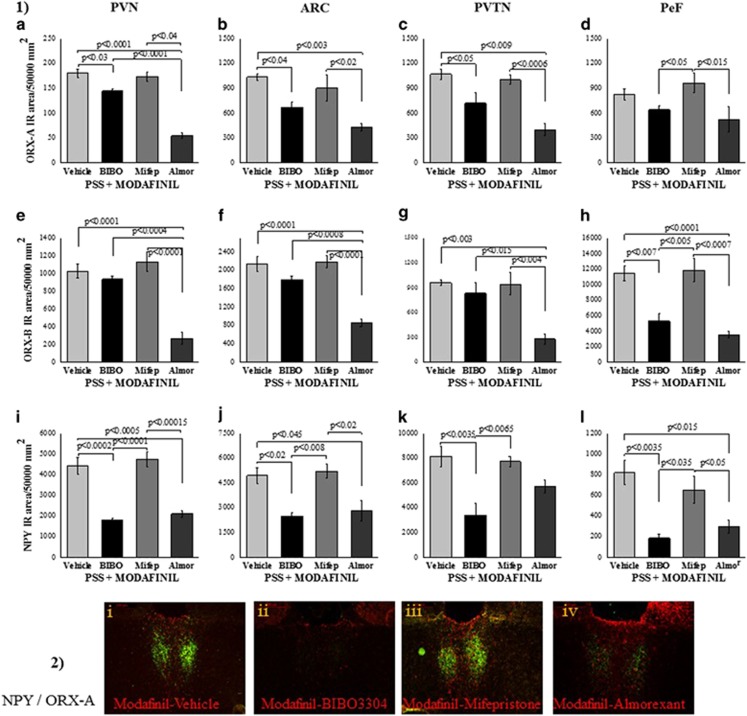
The NPY-Y1 receptor and the ORX receptors are necessary for the anxiolytic effects of modafinil: immunoreactivity of orexin-A, orexin-B and NPY in the hypothalamus. Rats were killed and their brains were removed for immunoreactivity analysis 2 h following the modafinil or vehicle injunctions, as depicted in the figure. (1) Quantitative morphometric analysis of ORX-A (**a–d**), ORX-B (**e–h**) and NPY (**i–l**) immunoreactivity in fibers and cells of the PVN (**a**, **e**, **i**), ARC (**b**, **f**, **j**), PVTN (**c**, **g**, **k**) and PeF (**d**, **h**, **l**) in rats treated with vehicle (*n*=4), mifepristone (*n*=4), BIBO3304 (*n*=4) or almorexant (*n*=4), exposed to predator-scent stress (PSS), and then, 30 min later, treated with modafinil. (2) Representative images of ORX-A and NPY immunoreactivity in the PVTN of PSS-exposed rats treated with either vehicle (i), BIBO3304 (ii), mifepristone (iii) or almorexant (iv) before modafinil injection. Images were acquired at a × 10 magnification. Scale bar, 200 μm. The cells in green are NPY-positive and the cells in red are ORX-A-positive. Almorexant and BIBO3304 downregulated the immunoreactivity of ORX-A, ORX-B and NPY fibers and cells in the PVN (F(3,12)=61.1, *P*<0.0001; F(3,12)=25.4, *P*<0.0002; and F(3,12)=29.9, *P*<0.000, respectively), ARC (F(3,12)=8.7, *P*<0.0025; F(3,12)=26.3, *P*<0.0001; and F(3,12)=9.3, *P*<0.002, respectively), PVTN (F(3,12)=13.8, *P*<0.0004; F(3,12)=10.2, *P*<0.0015; and F(3,12)=9.2, *P*<0.002, respectively) and PeF (F(3,12)=3.5, *P*<0.05; F(3,12)=16.9, *P*<0.00015; and F(3,12)=9.5, *P*<0.002, respectively). BIBO, BIBO3304; Mifep, mifepristone; Almor, almorexant; PVN, paraventricular nucleus; ARC, arcuate hypothalamic nucleus; PVTN, paraventricular thalamic nucleus; PeF, perifornical reign of lateral hypothalamus; ORX, orexin; NPY, neuropeptide Y. Bars represent group means±s.e.m.

To elucidate the molecular changes that are coupled with these pharmacological manipulations, we treated another batch of rats as described above, but collected their brains 2 h following exposure to PSS. These experiments revealed that, as compared with vehicle, both almorexant and BIBO3304 downregulated the immunoreactivity of ORX-A, ORX-B and NPY fibers and cells in a region-specific manner. Thus, the almorexant pre-injection significantly reduced the density of ORX-A-immunoreactive, ORX-B-immunoreactive and NPY-immunoreactive in cells and fibers of the PVN (*P*<0.0001, *P*<0001 and *P*<0.0005, respectively; Figures 4a, e and i) and ARC (*P*<0.003, *P*<0.0001 and *P*<0.045, respectively; Figures 4b, f and j), the density of ORX-A-immunoreactive and ORX-B-immunoreactive in cells and fibers of the PVTN (*P*<0.009 and *P*<0.003, respectively; Figures 4c and g) and the density of ORX-B-immunoreactive and NPY-immunoreactive in cells and fibers of the PeF (*P*<0.0001 and *P*<0.015, respectively; Figures 4h and l). Similarly, the BIBO3304 pre-injection significantly reduced the density of ORX-A-immunoreactive and NPY-immunoreactive in cells and fibers of the PVN (*P*<0.03 and *P*<0.0002, respectively; Figures 4a and i), ARC (*P*<0.04 and *P*<0.02, respectively; Figures 4b and j) and PVTN (*P*<0.04 and *P*<0.05, respectively; Figures 4c and k) and the density of ORX-B-immunoreactive and NPY-immunoreactive in cells and fibers of the PeF (*P*<0.007 and *P*<0.0035, respectively; Figures 4h and l). These results confirm that activation of the NPY and ORX systems are required for the behavioral anxiolytic response to modafinil following PSS exposure

### Time course of the effects of PSS exposure on ORX-immunoreactive and NPY-immunoreactive in the hypothalamus

**Figure 5:**
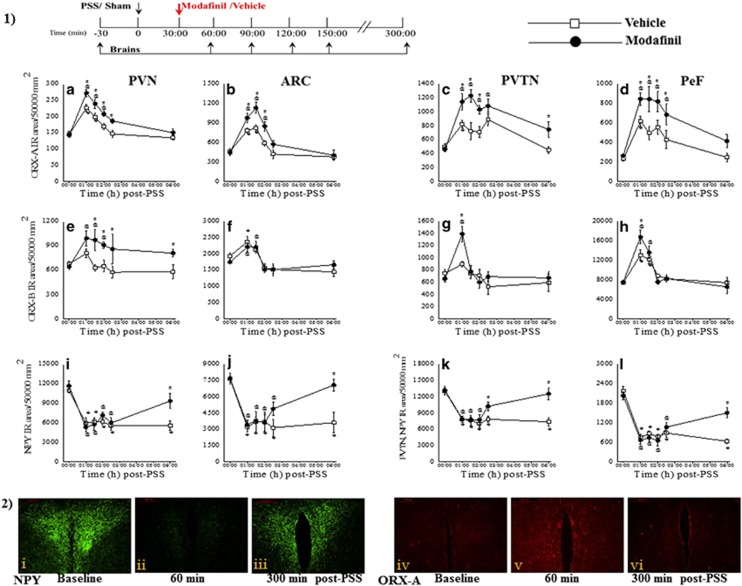
Time course of the effects of PSS exposure on ORX and NPY immunoreactivity in the hypothalamus. The experimental protocol is depicted in the top panel. The rats were exposed for 10 min to predator-scent stress (PSS) or to Sham-PSS and then, 30 min later, they were injected intraperitoneally with either modafinil (350 mg kg^−1^) or vehicle. The expression of ORX-A, ORX-B and NPY was examined in the hypothalamus before or 30, 60, 90, 120 or 330 min following the post-PSS modafinil or vehicle treatment (*n*=62 overall, five to six rats at each time point). (1) Quantitative morphometric analysis of ORX-A (**a**–**d**), ORX-B (**e**–**h**) and NPY (**i**–**l**) immunoreactivity in fibers and cells of the PVN (**a**, **e**, **i**), ARC (**b**, **f**, **j**), PVTN (**c**, **g**, **k**) and PeF (**d**, **h**, **l**). (2) Representative images of NPY (left panels) and ORX-A (right panels) immunoreactivity in the PVN. The rats were exposed to PSS and treated with modafinil 30 min later. The brains were removed and analyzed either 30 min before PSS exposure (‘baseline') or 60 or 300 min after the modafinil treatment. Images were acquired at a × 10 magnification. Scale bar, 200 μm. The cells in green are NPY-positive and the cells in red are ORX-positive. A significant increase in ORX-A and ORX-B immunoreactivity in cells and fibers of the hypothalamus were observed 2 h after the exposure to PSS in both vehicle- and modafinil-treated rats (two-way analysis of variance (ANOVA): PVN: Orexin-A: treatment (main effect): F(1,50)=27.8, *P*<0.0001; time (main effect): F(5,50)=37.6, *P*<0.0001. Orexin-B: treatment (main effect): F(1,50)=19.9, *P*<0.0001; ARC: Orexin-A: treatment (main effect): F(1,50)=20.6, *P*<0.0001, time: F(5,50)=34.5, *P*<0.0001, treatment × time interaction: F(5,50)=2.5, *P*<0.05. Orexin-B: time (main effect): F(5,50)=14.4, *P*<0.001; PVTN: Orexin-A: treatment (main effect): F(1,50)=32.5, *P*<0.0001, time (main effect): F(5,50)=15.7, *P*<0.0001, treatment × time interaction: F(5,50)=2.7, *P*<0.035. Orexin-B: time (main effect): F(5,50)=8.4, *P*<0.0001, treatment × time interaction: F(5,50)=2.7, *P*<0.035; PeF: Orexin-A: treatment (main effect): F(1,50)=25.5, *P*<0.0001, time (main effect): F(5,50)=15.7, *P*<0.0001. Orexin-B: time (main effect): F(5,50)=29.7, *P*<0.0001). Following exposure to PSS, a significant decrease in NPY immunoreactivity in cells and fibers in the hypothalamus was observed in both vehicle- and modafinil-treated rats in the PVN (two-way ANOVA: treatment: NS, time: F(5,50)=20.2, *P*<0.0001, treatment × time interaction: F(5,50)=2.4, *P*<0.05), ARC (treatment: F(1,50)=6.0, *P*<0.02, time: F(5,50)=14.3, *P*<0.0001, treatment × time interaction: F(5,50)=2.4, *P*<0.05), PVTN (treatment: F(1,50)=10.3, *P*<0.003, time: F(5,50)=17.0, *P*<0.0001, treatment × time interaction: F(5,50)=3.4, *P*<0.015) and PeF (treatment: NS, time: F(5,50)=39.2, *P*<0.0001, treatment × time interaction: F(5,50)=4.9, *P*<0.001). However, modafinil treatment significantly increased NPY-immunoreactive in the PVN and ARC areas 6 h following the exposure. BIBO, BIBO3304; Mifep, mifepristone; Almor, almorexant; PVN, paraventricular nucleus; ARC, arcuate hypothalamic nucleus; PVTN, paraventricular thalamic nucleus; PeF, perifornical reign of lateral hypothalamus; ORX, orexin; NPY, neuropeptide Y. Bars represent group means±s.e.m.

To elucidate the molecular changes that occur immediately following PSS and modafinil treatment, we examined the expression of ORX-A, ORX-B and NPY in the hypothalamus before and 30, 60, 90, 120 or 330 min following the post-PSS modafinil or vehicle treatment (Figure 5). Regardless of the treatment regimen, all the rats showed a marked increase in the density of ORX-A-immunoreactive and ORX-B-immunoreactive cells and fibers 30 min after the modafinil treatment, followed by a decline that lasted about 6 h. However, the density of ORX-A-immunoreactive and ORX-B-immunoreactive in the PVN, ARC, PVTN and PeF was significantly higher in modafinil-treated rats than in vehicle-treated rats (*P*<0.025 for each of these regions; Figures 5a–d). These results suggest that endogenous ORXs are released in response to (or during) exposure to PSS and in response to modafinil treatment. Overall, the increases in ORX expression in the hypothalamus may be associated with increased vigilance—a trait associated with anxiety,^24^ that is, with the acute response to stress.

The magnitude of response to PSS in the NPYergic system was different than that of the ORXs: following exposure to PSS, a significant decrease in NPY-immunoreactive cells and fibers in the hypothalamus (as compared with baseline levels) was observed in both vehicle- and modafinil-treated rats. However, modafinil-treated rats displayed an increase in NPY-immunoreactive in the PVN and ARC 6 h following the exposure, whereas no change was observed in the vehicle-treated rats. This finding may suggest that, in the hypothalamus, the modafinil-induced changes in NPY expression may commence as early as 3 h after the administration of modafinil, or that the NPYergic system is not a direct pharmacological target of modafinil. Therefore, we postulate that there are other neurotransmitters through which modafinil acts on the NPY system.

## Discussion

We report that a single dose of modafinil (350 mg kg^−1^), injected 30 min after PSS exposure, significantly moderated behavioral patterns that represent stress-induced anxiety, avoidance and hyperarousal in the EPM and ASR paradigms. Modafinil completely prevented PTSD-like phenotypes in rats exposed to the traumatic stress and, concomitantly, increased the minimal and partial response patterns, thus reflecting a significant overall shift toward a more adaptive behavioral response to stress. A similar pattern of behavioral stress responses was observed when rats were sleep-deprived by gentle handling for 6 h following PSS exposure,^7^ suggesting that the effects of modafinil were mediated by the post-exposure wakefulness.

We found that modafinil upregulated endogenous hypothalamic ORXs shortly after administration. As expected, the activation of hypothalamic ORX neurons by PSS or by modafinil resulted in hyperlocomotion or increased wakefulness,^25^ as ORX-containing neurons are critical components of the circuitry that regulates and determines the arousal threshold.^26^ Arousal is necessary for the initial response to threats and, therefore, it is a key component in the stress response. It has been proposed that the ORXergic system is an important component of the central response that leads to adaptive stress responses.^24^

Although PSS exposure immediately downregulated hypothalamic NPY-immunoreactive density in all the rats, modafinil upregulated NPY in the hypothalamus, beginning 2.5 h after injection and peaking after approximately 6 h. This upregulation was not observed in vehicle-treated PSS-exposed rats. The marked upregulation of ORXs and NPY in the hypothalamic nuclei was observed 8 days after a single dose of modafinil, and it remained stable in the longer term. This finding is consistent with several lines of evidence attesting to the resilience-promoting properties of NPY.^18, 27^ Confirming these results, we found that post-stressor administration of modafinil was ineffective in attenuating the stressor-induced behavioral disruption when rats were pre-injected with BiBO3304 or with almorexant, and this effect was mediated, to a great extent, by the ORX and NPY-Y1 receptors.

The mechanism of action of modafinil is still debated,^28, 29, 30^ and various theories have been proposed, including direct activation of ORX neurons.^31^ It is plausible that modafinil acts, either directly or indirectly, both on NPY-Y1 receptors and on ORX receptors. The finding that hypothalamic ORX levels are upregulated immediately after modafinil administration, whereas NPY levels increase at a later time suggest an interaction or cross-talk between the two systems; it is even possible (although not directly addressed in this study) that modafinil activates the NPYergic system via the ORXergic system. Indeed, there is evidence of interactions between hypothalamic ORXs and NPY, as the two systems are co-localized in hypothalamic neurons,^32, 33^ which integrate and transmit the stress signals to both central and peripheral regions^34^ to affect feeding behavior, energy homeostasis, cardiovascular regulation and endocrine functions.^35, 36^ These assumptions are supported by the fact that almorexant—a dual orexin receptor antagonist—disrupted not only the hypothalamic ORXs expression, but also the hypothalamic NPY expression. Similarly, the NPY-Y1 receptor antagonist BiBO3304 disrupted not only hypothalamic NPY levels, but also hypothalamic ORXs expressions—a finding with pivotal implications, as it indicates that the cross-talk between ORXs and NPY may be necessary for the anxiolytic effects of modafinil-induced wakefulness. Taken together, the combined upregulation of hypothalamic ORX and NPY neurons may have a key role in promoting wakefulness and mediate the anxiolytic-like effects of modafinil.

We found that modafinil elevates circulating corticosterone levels under both control and PSS conditions. Our previous studies highlight the importance of an initial bolus of endogenous or exogenous corticosteroids in the adaptive response to stress and in the return to homeostasis,^37, 38, 39^ and the increase in corticosterone levels following modafinil treatment thus appear to contribute to an adaptive response.

Blocking endogenous GCs signaling by mifepristone—a classic antagonist of intracellular glucocorticoid receptors—at a pharmacologically active dose did not affect the anxiolytic effect of modafinil on day 7. As reported previously,^40^ it is possible that GR antagonists interfere with glucocorticoid negative feedback and increase corticosterone, thereby activating mineralocorticoid receptors in the brain and reducing the efficacy of antagonism at relevant sites.^40^ Alternatively, the mechanism by which modafinil attenuates the stress-related sequelae could involve activation of the HPA axis through non-genomic signaling,^41, 42^ either via membrane-associated variants of the classical receptors^43^ or via cytoplasmic receptors,^44^ or through transcriptional signaling, which depends on interaction with other transcription factors (AP-1 and NF-κB).^40^ In addition, it is possible that the anxiolytic effects of modafinil do not depend directly or solely on the HPA axis. Stimulation of the HPA axis could be involved in the behavioral effects of modafinil in concert with the ORX or NPY systems.

The neuroendocrine stress response has also been reported to involve the ORX system.^45, 46, 47, 48^ Thus, the ORXergic system, activated by stress, likely acts on different targets. At the hypothalamic level, ORX may activate the HPA axis by increasing the release of CRF in the median eminence.^49^ In addition, ORXs exert a selective and direct glucocorticoid secretagogue activity on the rat adrenals, acting through a receptor-mediated activation of the adenylate cyclase/PKA-dependent signaling pathway.^50, 51, 52, 53^ Thus, we suggest that modafinil-induced endogenous upregulation of ORX levels in the hypothalamic nuclei may be involved in the activation of the HPA axis. Moreover, we previously reported that the NPYergic system, together with the HPA axis, has a significant role in conferring resilience to stress.^18, 27^ Although the direct involvement of the HPA axis in the anxiolytic-like effects of modafinil has not been demonstrated previously, we believe that ORX release following modafinil treatment stimulates the HPA axis. Alternatively, ORXs may stimulate the HPA axis, at least in part, via the NPY-Y1 receptor. Our results suggest that modafinil has a role in the maintenance of homeostasis in response to PSS through several mechanisms, including an increase in ORX release, an agonistic effect on the NPY-Y1 receptor, and modulation of the HPA activity and reactivity.

To date, there is no consensus regarding the effect of modafinil on circulating corticosterone levels. Although some pre-clinical and clinical studies have reported that modafinil increases basal and stress-induced corticosterone levels,^54, 55^ others reported no difference in corticosterone levels.^56^

In summary, we show that modafinil administered during the first hours following exposure to stress may be a simple, but effective, intervention for the secondary prevention of stress-induced pathologies and may be beneficial in attenuating the traumatic stress-related sequelae. Mechanistically, our findings suggests that modafinil stimulates circuits within the hypothalamus that tie together the NPY, ORXs and HPA-axis systems, which have been shown independently to participate in adaptive stress responses. Modafinil treatment stimulates an endogenous activation of these three systems and provides an additional regulatory mechanism for the modulation of adaptive stress responses. Elucidating the molecular mechanisms underlying the anxiolytic effects of modafinil may improve our ability to design therapeutic interventions for stress-related disorders.
