# Supplementary material for: The wake-promoting drug modafinil stimulates specific hypothalamic circuits to promote adaptive stress responses in an animal model of PTSD
Source: Transl Psychiatry. 2016 Oct 11;6(10):e917–. doi: 10.1038/tp.2016.172 (PMC5315545; doi:10.1038/tp.2016.172)
Supplement: Supplementary Materials and Methods [file tp2016172x1.docx]

**SUPPLEMENTARY MATERIALS AND METHODS**

**Experimental design:**

To assess the behavioral and physiological responses to modafinil treatment in the aftermath of predator scent stress (PSS), four consecutive experiments were conducted, each stemming from the results of the preceding one. The experimental design used for each of these experiments is schematically depicted in the respective figures. In the first experiment (n=125), all rats were exposed to PSS or to sham-PSS (see below) and modafinil (350 mg/kg) or vehicle was injected intraperitoneally (i.p.) 30 min after exposure. Behavior was assessed on Day 7, first in the elevated plus maze (EPM) paradigm and then, 1 h later, in the acoustic startle reaction (ASR) paradigm. These data subsequently served for classification into behavioral response groups (see ^1, 2^). The rats were sacrificed on Day 8 and their brains were collected for measurement of NPY, ORX-A, and ORX-B immunoreactivity (see below) in four hypothalamic regions: the paraventricular nucleus (PVN), the arcuate nucleus (ARC), the paraventricular thalamic nucleus (PVTN), and the perifornical region of the lateral hypothalamus (PeF). In the second experiment (n=96), circulating corticosterone levels were evaluated before and at different time points (15-300 min) after modafinil (350 mg/kg) or vehicle treatment, administrated 30 min after exposing rats to PSS or to sham-PSS. The third experiment (n=111) was designed to evaluate the behavioral effects of modafinil treatment with and without pharmacologically manipulating NPY, ORXs, or GR levels prior to the PSS exposure. To this end, rats were injected with the NPY-Y1R antagonist BIBO3304, the dual ORX receptor antagonist almorexant, the GR antagonist mifepristone, or vehicle, in each case with or without modafinil. The injections were performed 10 min prior to or, for mifepristone, 5 min following exposure to PSS, and modafinil or vehicle were injected i.p. 30 min after exposure to PSS or Sham-PSS. To elucidate the molecular changes that are acutely coupled with these pharmacological manipulations, brains were collected 2 h following the exposure to PSS from 4 rats in each group. For the remaining rats, behavioral parameters were assessed on Day 7. In the last experiment (n=62), we examined the expression of ORX-A, ORX-B, and NPY in the hypothalamus either prior to or 30, 60, 90, 120, or 330 minutes following an i.p. injection of modafinil or vehicle.

We chose to focus on ORXs and NPY for several reasons. First, the ORX-ergic system, which regulates sleep–wake control^3, 4^, is one of the leading candidate system to be involved in the wake-promoting action of modafinil^5, 6^. Second, orexin neurons integrate a multitude of central and peripheral stress-related inputs, including the HPA axis, and are critical modulators or actuators in the neural circuitry of stress^7, 8^. Third, we have previously found that the NPY-ergic system plays a significant role in recovery from and resilience to stress^9, 10^, and recent data indicate a direct interaction between ORX and NPY in the hypothalamus, which may contribute to the central regulation of metabolic and endocrine processes in both rodents and primates^11^.

***The Cut-off Behavioral Criteria (CBC) model:*** Human responses to traumatic experiences clearly vary greatly in extent and in character. More importantly, PTSD occurs in a percentage (roughly 25–35%) of the individuals exposed to potentially traumatic events, underscoring the importance of contemporary definitions of stress-related disorders (DSM and ICD) and of inclusion and exclusion criteria applied in controlled clinical trials. In contrast, most animal studies have tended to relate to unclassified “global” groups, i.e., the entire exposed population versus control populations without distinction, whereas researchers who work with animals have long been aware that individual study subjects tend to display a variable range of responses to stimuli, certainly where stress paradigms are concerned. The heterogeneity in animal responses might be regarded as confirming the validity of animal studies, rather than as a problem. It stands to reason that a model of diagnostic criteria for psychiatric disorders can be applied to animal responses to augment the validity of study data, as long as the criteria for classification are clearly defined, reliably reproducible, and yield results that conform to findings in human subjects. The criteria used in this model were thus based on the EPM and ASR paradigms combined, and they clearly define two opposing extremes of the possible responses of each individual to stress. The one extreme of this model, termed here an ‘extreme behavioral response’ (EBR), indicates animals whose exploration of the open arms of the EPM was zero throughout the test, and whose startle response was maximal and did not undergo any habituation throughout the ASR test. An EBR thus parallels extreme PTSD-like responses and unabating maximal stress. The other extreme, termed here a ‘minimal behavioral response’ (MBR), indicates animals whose behavior in both the EPM and ASR paradigms was virtually unaffected by the stressor. A MBR thus parallels no PTSD-like response to the stressor. Rats that did not meet the criteria for either an EBR or a MBR were considered, by default, to have a partial behavioral response (PBR)^2, 12, 13^.

**Intranasal (IN) administration:** BIBO3304 (10 μg) or vehicle (distilled water) were infused into each nare by using a pipetteman with a disposable plastic tip under light isoflurane anesthesia. Extreme care was taken to avoid contact with the IN mucosa. Following the IN administration, the head of the animal was held in a tilted back position for approximately 10 s to prevent loss of solution from the nares**.**

**REFERENCES**

1. Cohen H, Matar MA, Joseph Z. Animal models of post-traumatic stress disorder. *Curr Protoc Neurosci* 2013; **Chapter 9:** Unit9 45.

2. Cohen H, Zohar J, Matar M. The relevance of differential response to trauma in an animal model of posttraumatic stress disorder. *Biol Psychiatry* 2003; **53**(6)**:** 463-473.

3. Espana RA, Baldo BA, Kelley AE, Berridge CW. Wake-promoting and sleep-suppressing actions of hypocretin (orexin): basal forebrain sites of action. *Neuroscience* 2001; **106**(4)**:** 699-715.

4. Mieda M, Yanagisawa M. Sleep, feeding, and neuropeptides: roles of orexins and orexin receptors. *Current opinion in neurobiology* 2002; **12**(3)**:** 339-345.

5. Ishizuka T, Murotani T, Yamatodani A. Modanifil activates the histaminergic system through the orexinergic neurons. *Neurosci Lett* 2010; **483**(3)**:** 193-196.

6. Scammell TE, Estabrooke IV, McCarthy MT, Chemelli RM, Yanagisawa M, Miller MS*, et al*. Hypothalamic arousal regions are activated during modafinil-induced wakefulness. *The Journal of neuroscience : the official journal of the Society for Neuroscience* 2000; **20**(22)**:** 8620-8628.

7. Berridge CW, Espana RA, Vittoz NM. Hypocretin/orexin in arousal and stress. *Brain Res* 2010; **1314:** 91-102.

8. Yamanaka A, Beuckmann CT, Willie JT, Hara J, Tsujino N, Mieda M*, et al*. Hypothalamic orexin neurons regulate arousal according to energy balance in mice. *Neuron* 2003; **38**(5)**:** 701-713.

9. Cohen H, Liu T, Kozlovsky N, Kaplan Z, Zohar J, Mathe AA. The neuropeptide Y (NPY)-ergic system is associated with behavioral resilience to stress exposure in an animal model of post-traumatic stress disorder. *Neuropsychopharmacology* 2012; **37**(2)**:** 350-363.

10. Cohen S, Vainer E, Matar MA, Kozlovsky N, Kaplan Z, Zohar J*, et al*. Diurnal fluctuations in HPA and neuropeptide Y-ergic systems underlie differences in vulnerability to traumatic stress responses at different zeitgeber times. *Neuropsychopharmacology* 2015; **40**(3)**:** 774-790.

11. Horvath TL, Diano S, van den Pol AN. Synaptic interaction between hypocretin (orexin) and neuropeptide Y cells in the rodent and primate hypothalamus: a novel circuit implicated in metabolic and endocrine regulations. *The Journal of neuroscience : the official journal of the Society for Neuroscience* 1999; **19**(3)**:** 1072-1087.

12. Cohen H, Matar MA, Joseph Z. Animal models of post-traumatic stress disorder. *Curr Protoc Neurosci* 2013; **Chapter 9:** Unit 9 45.

13. Cohen H, Zohar J, Matar MA, Kaplan Z, Geva AB. Unsupervised fuzzy clustering analysis supports behavioral cutoff criteria in an animal model of posttraumatic stress disorder. *Biol Psychiatry* 2005; **58**(8)**:** 640-650.
