# Supplementary material for: The wake-promoting drug modafinil stimulates specific hypothalamic circuits to promote adaptive stress responses in an animal model of PTSD
Source: Transl Psychiatry. 2016 Oct 11;6(10):e917–. doi: 10.1038/tp.2016.172 (PMC5315545; doi:10.1038/tp.2016.172)
Supplement: Supplementary Figures [file tp2016172x2.docx]

**Supplementary Fig S1.**

**
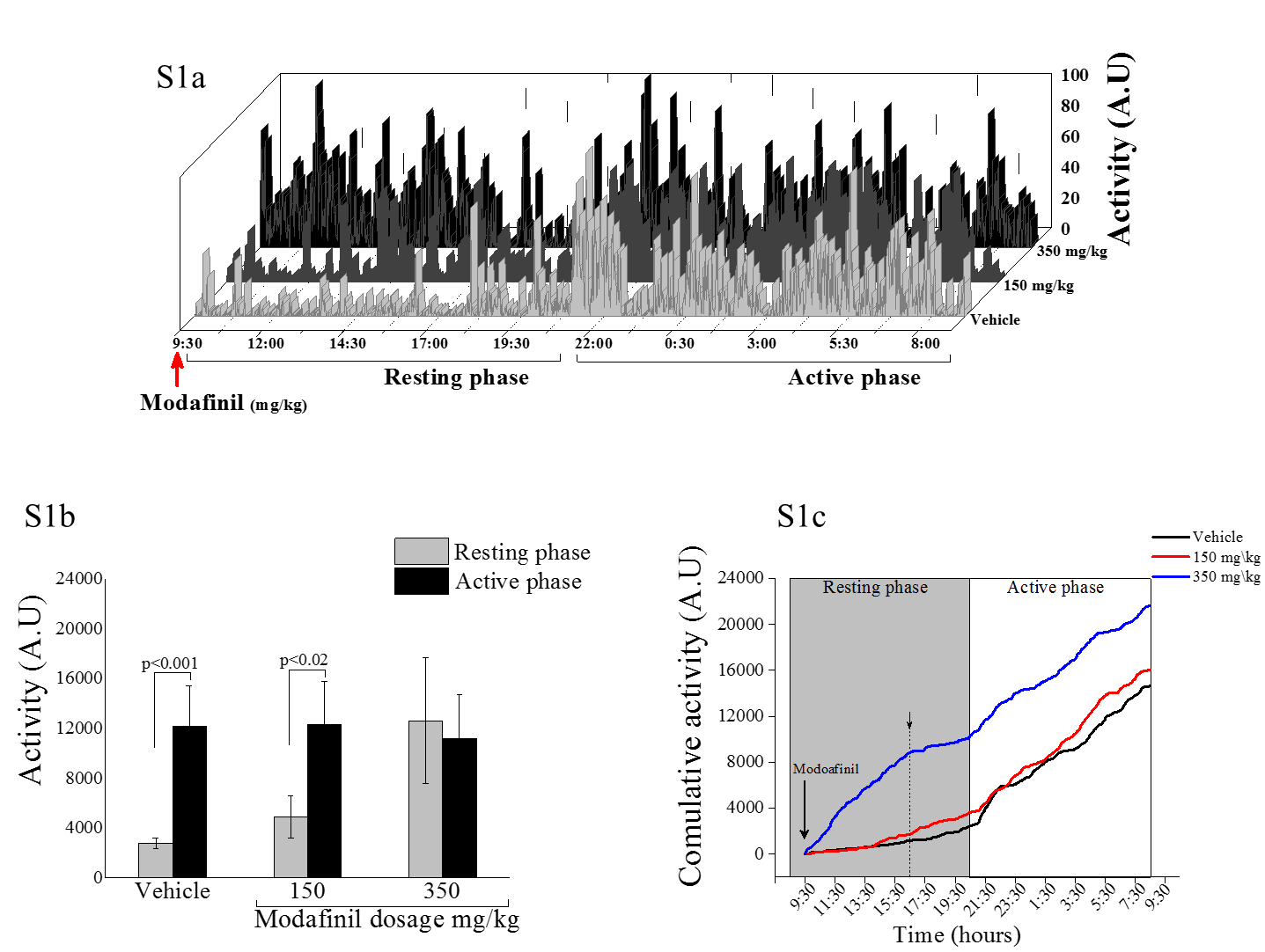
**

**Fig. S1: Modafinil reversibly promotes wakefulness in rats**

After 24h of baseline locomotor activity (LMA), rats were i.p. injected with 150 or 350 mg/kg modafinil or vehicle (n=4 rats in each group) during the resting phase of their 12:12-h light-dark cycle, and their LMA was continuously recorded throughout the next 24 h by using an automated system. (A) LMA pharmaco-dynamic profiles during the initial 24 h after treatment with modafinil or vehicle. (B) LMA counts. A significant main effect was found for Treatment [repeated measures ANOVA: F(2,9)=8.98, p<0.008], such that, when administered 350 mg/kg modafinil, but not 150 mg/kg modafinil or vehicle alone, LMA was significantly increased immediately following administration. (C) The cumulative duration of LMA in rats treated with 350 mg/kg modafinil appeared to subside completely within 6 h. Data represent group mean ± S.E.M

**Experimental design:** After a 10-d adaptation period, the locomotor activity was sampled for 24 h to collect baseline rhythms.

**Radiotelemetry system:** The radiotelemetry system employed in this study enabled recording of the locomotor activity from freely moving rats. Care was taken to ensure that the rats did not experience unnecessary pain or distress during the procedures.

**Telemetric transmitter implantation:** Wireless radiofrequency transmitters (Data Sciences International (DSI), St. Paul, MN; model TA10ETA-F20) were implanted for continuous locomotor activity recordings under aseptic conditions during the light period, employing previously described procedures^1, 2^. Briefly, the rats were intraperitoneally anesthetized with ketamine (60-80 mg/kg) and xylazine (5-10 mg/kg) and the transmitters were implanted intraperitoneally. The body of the transmitter was placed inside the abdominal cavity and the two electrodes (wire loops) were fixed to the dorsal surface of the xiphoid process and in the anterior mediastinum close to the right atrium. The leads were directed rostrally subcutaneously (sc) and anchored in place with permanent sutures (DII positioning). The rats were prophylactically injected with penicillin (natrium-penicillin G, 40,000 IU/kg body weight, sc; Hanford's United States Veterinary Products) 10-15 min prior to the incision, and codeine (1 mg/100 ml) was added to their drinking water for 3 days after the procedure. After the surgical procedures, all animals were housed in custom-designed divided cages for 5 days to permit adequate healing of the suture wounds ^3^ and they were then returned to the home cages to recover for an additional 5 days.

**Data acquisition and analysis:** Radio-telemetric recordings**:** The telemetry data were recorded with a radio-telemetry receiver (DSI, St. Paul, MN; sampling rate 1 kHz, 12-bit precision digitizing). The radiotelemetry receiver was controlled by the vendor’s software (Dataquest ART, Version 4.1 Acquisition software; DSI, St. Paul, MN).

**REFERENCES**

1. Grippo A, Lamb D, Carter C, Porges S. Cardiac regulation in the socially monogamous prairie vole. *Physiol Behav* 2007; **90:** 386-393.

2. Sgoifo A, Stilli D, Medici D, Gallo P, Aimi B, Musso E. Electrode positioning for reliable telemetry ECG recordings during social stress in unrestrained rats. *Physiol Behav* 1996; **60:** 1397-1401.

3. Grippo AJ, Lamb DG, Carter CS, Porges SW. Cardiac regulation in the socially monogamous prairie vole. *Physiol Behav* 2007; **90**(2-3)**:** 386-393.

**Supplementary Fig S2.**


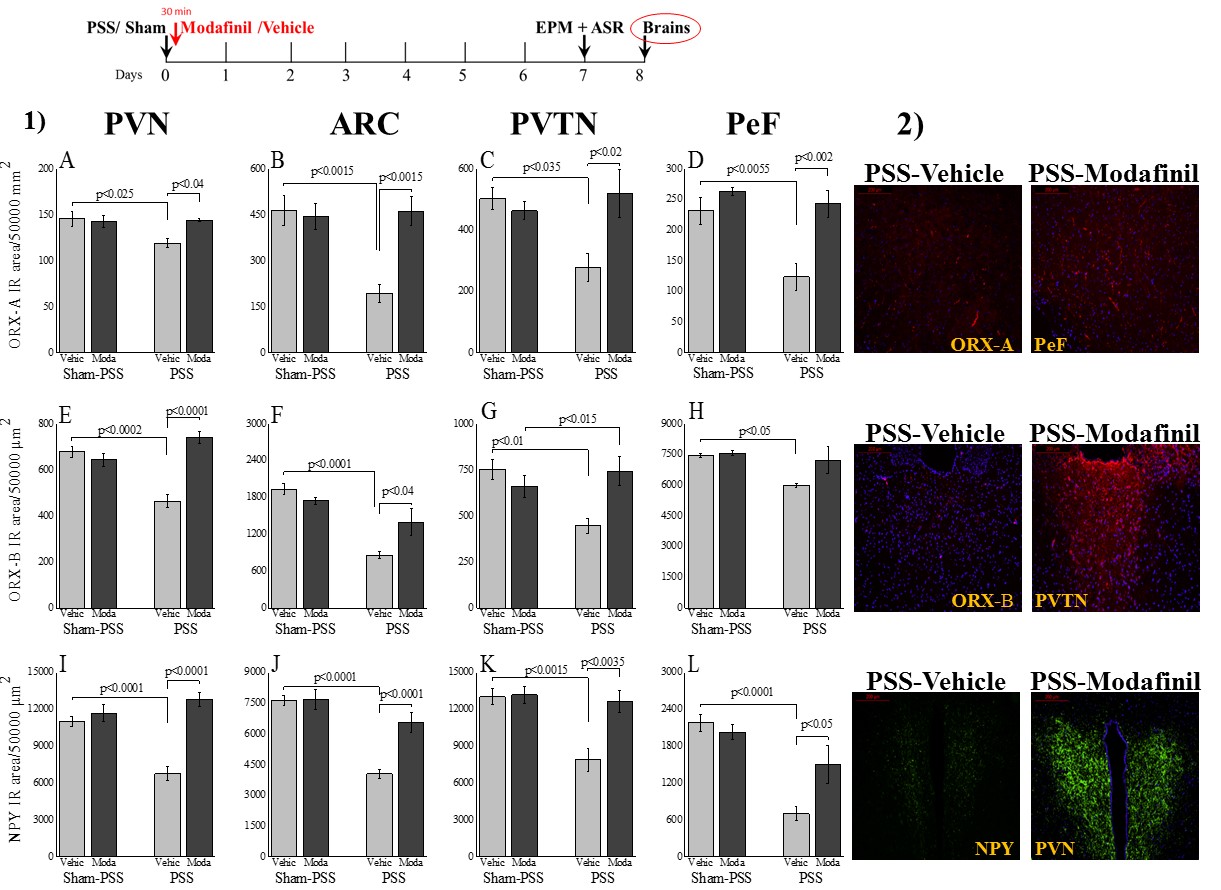


**Fig S2:** **Modafinil increases the immunoreactivity of orexin-A, orexin-B, and NPY in the hypothalamus**

The experimental protocol is shown on the top panel, and is similar to that described in Fig. 1. 1) A quantitative morphometric analysis of the immunoreactivity of ORX-A (A–D), ORX-B (E–H) and NPY (I–L) in fibers and cells of the PVN (A, E, I), ARC (B, F, J), PVTN (C, G, K) and PeF (D, H, L) in rats exposed to either predator-scent stress (PSS) or Sham-PSS and treated with either vehicle or modafinil (n=6 for each of the four groups). 2) Representative immunoreactivity images of ORX-A in the PeF (top), ORX-B in the PVTN (middle), and NPY in the PVN (bottom) of rats exposed to PSS and treated with either vehicle (left panels) or modafinil (right panels). Images were acquired at a 10× magnification. Scale bar: 200 μm. The cells in red are ORX-positive and the cells in green are NPY-positive.

Modafinil administration, as compared with vehicle administration, significantly upregulates ORX-A and NPY immunoreactivity in cells and fibers of the PVN, ARC, PVTN, and PeF [two-way ANOVA: Exposure × Treatment interaction; PVN: F(1,20)=5.98, p<0.025 and F(1,20)=21.9, p<0.00015, respectively; ARC: F(1,20)=11.5, p<0.003 and F(1,20)=10.4, p<0.0045, respectively; PVTN: F(1,20)=7.6, p<0.015 and F(1,20)=25.8, p<0.00015, respectively; PeF: F(1,20)=5.0, p<0.04 and F(1,20)=57.1, p<0.00015, respectively]. In addition, modafinil treatment, as compared with vehicle treatment, significantly upregulates ORX-B immunoreactivity in cells and fibers of the PVN and ARC regions [two-way ANOVA: Exposure × Treatment interaction: PVN: F(1,20)=30.8, p<0.0001; ARC: F(1,20)=8.55, p<0.0085)].

PVN – paraventricular nucleus; ARC – arcuate hypothalamic nucleus; PVTN – paraventricular thalamic nucleus; PeF – perifornical reign of lateral hypothalamus; ORX – orexin; NPY – neuropeptide Y.

Bars represent group means ± S.E.M
